# Bone microarchitecture deteriorations and a fragility fracture in a patient with beta and alpha heterozygous thalassemia: a case report

**DOI:** 10.1007/s00508-016-1032-7

**Published:** 2016-06-30

**Authors:** Xaver Feichtinger, Roland Kocijan, Heinrich Resch, Christian Muschitz

**Affiliations:** Medical Department II, St. Vincent Hospital Vienna, Academic Teaching Hospital of the Medical University Vienna, Stumpergasse 13, 1060 Vienna, Austria

**Keywords:** Bone microarchitecture, HR-pQCT, Thalassemia

## Abstract

To date there are few studies that have investigated bone mineral density (BMD) and markers of bone metabolism in patients with thalassemia minor form. None of the previous trials presented bone structure analysis in the patient populations. We present the case of a 24-year-old Turkish woman with heterozygous beta and alpha thalassemia who sustained a low-trauma fracture of the inferior pubic ramus. Despite normal markers of bone metabolism, the dual X‑ray absorptiometry (DXA) showed decreased areal bone mineral density. Furthermore, severely reduced bone structure parameters and reduced volumetric bone mineral density was assessed by high-resolution peripheral quantitative computed tomography (HR-pQCT). Due to these diagnostic findings at time of peak bone mass, an osteoanabolic therapy with teriparatide for 24 months was initiated. The findings concerning BMD and bone structure in this patient can be seen as caused by the beta and alpha thalassemia.

## Introduction

Thalassemias name a group of hereditary diseases of hemoglobin synthesis due to a defect in production of one or more of the globin chains of hemoglobin [1]. In more severe forms of thalassemia, ineffective erythropoiesis necessitates transfusion therapy [2]. Reduction in bone mineral density (BMD), increased fractures, deformity and chronic bone pain are well known problems in patients with beta thalassemia major [3–6]. To date there are few studies that have investigated BMD and markers of bone metabolism in patients with thalassemia minor [7, 8]. None of the previous trials demonstrated bone structure analysis in these patients. We present the case of a patient with bone structure deterioration and a low-trauma fracture with heterozygous beta and alpha thalassemia as underlying diseases.

## Case report

A 24-year-old Turkish woman with known non-transfusion dependent thalassemia was admitted to a specialized trauma center due to an undisplaced low-traumatic fracture of the inferior pubic ramus on the right side. The trauma was sustained when the patient was walking downstairs. First treatment involved an analgesic therapy, anticoagulation therapy and early mobilization with progress in weight-bearing strength.

To better understand the cause of the fracture the patient was admitted to a specialized bone center. The patient’s medical history revealed no signs of secondary osteoporosis or endocrinological disorders. The family anamneses further revealed beta thalassemia of both the patient’s mother and her grandmother with no fractures in their respective medical histories. Sex hormones as well as gonadotropin levels were clarified before admission to the bone center and were within normal range. She neither had received any chelation therapy nor any hormone supplementation. The patient had no births and her menstrual cycle was normal and regular. The hemoglobin electrophoreses test as well as genetic testing for thalassemia was conducted. The results showed that the patient is heterozygous for both beta thalassemia and alpha thalassemia (‑α^3.7^/aa deletion mutation).

On clinical examination, a reduced BMI of 15.2 kg/m^2^ (weight 42 kg; height 166 cm) was observed. Due to a transient depressive period the patient lost 6 kg (from BMI 17.4 to 15.2 kg/m^2^) and regained weight again after several months. Psychiatric and dietetic examinations investigating a possible eating disorder did not show any indications of diseases such as anorexia and bulimia. Furthermore, the investigations demonstrated a well-balanced and calcium-rich dietary behavior.

### Laboratory values

Laboratory investigations (fasting, before 10 am) demonstrated high levels of erythrocytes and iron with reduced values of hemoglobin, MCV, MCH, MCHC and 25-hydroxyvitamin D. Serum values reflecting bone metabolism including procollagen aminoterminal propeptide type I (PINP, osteoblast activity), calcium, phosphate, intact parathyroid hormone (iPTH), type-1 collagen crosslinked C‑telopeptide (CTX, osteoclast activity) levels were all in normal range (Table 1).

**Table 1:** Laboratory values, dual X‑ray absorptiometry and high-resolution peripheral quantitative computed tomography (*HR-pQCT*) measurement results. HR-pQCT measurements are in comparison to a healthy control population [13]. Values out of normal range are bold.

Parameters (unit)	Value	Reference range
*Laboratory values*		
RBC (10^12^/l)	*5.78*	4.2–5.4
Hemoglobin (g/dl)	*11.1*	12–16
Hematocrit (%)	37.0	37–47
MCV (fl)	*64.0*	80–99
MCH (pg)	*19.3*	26.0–34.0
MCHC (g/dl)	*30.2*	32.0–36.0
RDW (%)	17.2	10.0–18.0
Platelets (10^9^/l)	214	150–370
Total serum iron (µg/dl)	*33.0*	37.0–145.0
Ferritin (ng/ml)	13.0	5–204
TSH (μU/ml)	1.70	0.40–4.00
Calcium (mmol/l)	2.51	2.10–2.58
Phospate (mmol/l)	1.17	0.60–1.55
PTH (pg/ml)	16.9	14.8–83.1
25-OH vitamin D (ng/ml)	*18.9*	≥20.0
Type-1 collagen crosslinked C‑telopeptide (CTX, ng/ml)	0.37	0.112–0.738
Procollagen type 1 (PINP, µg/l)	63.3	27.7–127.6
*BMD-DXA*		
*g/cm* ^2^		
L1–L4	0.809	
Femoral neck	0.582	
Total hip	0.592	
*Z-score*		
L1–L4	*−2.4*	
Femoral neck	*−2.9*	
Total hip	*−2.9*	
*T-score*		
L1–L4	*−3.1*	
Femoral neck	*−3.3*	
Total hip	*−3.4*	
*HR-pQCT (tibia)*		*Healthy controls; median (interquartile range) *[13]
Total BMD (mgHA/cm^3^)	*137.7*	305.3 (270.2, 347.3)
Trabecular BMD (mgHA/cm^3^)	*39.40*	169.3 (155.0, 200.7)
Cortical BMD (mgHA/cm^3^)	885.3	874.9 (832.0, 902.7)
BV/TV	*0.033*	0.141 (0.130, 0.170)
Tb.N (l/mm)	*0.650*	1.760 (1.590, 2.080)
Tb.Th (mm)	*0.050*	0.081 (0.074, 0.087)
Tb.1/N.SD (mm)	*1.606*	0.221 (0.170, 0.242)
Cortical thickness (mm)	*0.650*	1.130 (0.990, 1.410)
*HR-pQCT (radius)*		
Total BMD (mgHA/cm^3^)	297.3	325.7 (291.4, 386.3)
Trabecular BMD (mgHA/cm^3^)	*86.30*	160.4 (149.2, 190.0)
Cortical BMD (mgHA/cm^3^)	*929.1*	879.5 (849.5, 903.4)
BV/TV	*0.072*	0.129 (0.122, 0.158)
Tb.N (l/mm)	*1.350*	1.920 (1.760, 2.230)
Tb.Th (mm)	*0.053*	0.075 (0.065, 0.086)
Tb.1/N.SD (mm)	*0.319*	0.186 (0.158, 0.210)
Cortical thickness (mm)	0.790	0.775 (0.685, 0.878)

*RBC* red blood cell count, *MCV* mean corpuscular volume, *MCH* mean corpuscular hemoglobin, *MCHC* mean corpuscular hemoglobin concentration, *RDW* red cell distribution width, *TSH* thyroid-stimulating hormone, *PTH* parathyroid hormone, *BMD* bone mineral density, *DXA* dual energy X‑ray absorptiometry, *BV/TV* trabecular bone volume, *Tb.N* number of trabeculae, *Tb.Th* trabecular thickness, *Tb.1/N.SD* inhomogeneity of the trabecular network

### Areal BMD, volumetric BMD and bone microarchitecture

**Fig. 1:**
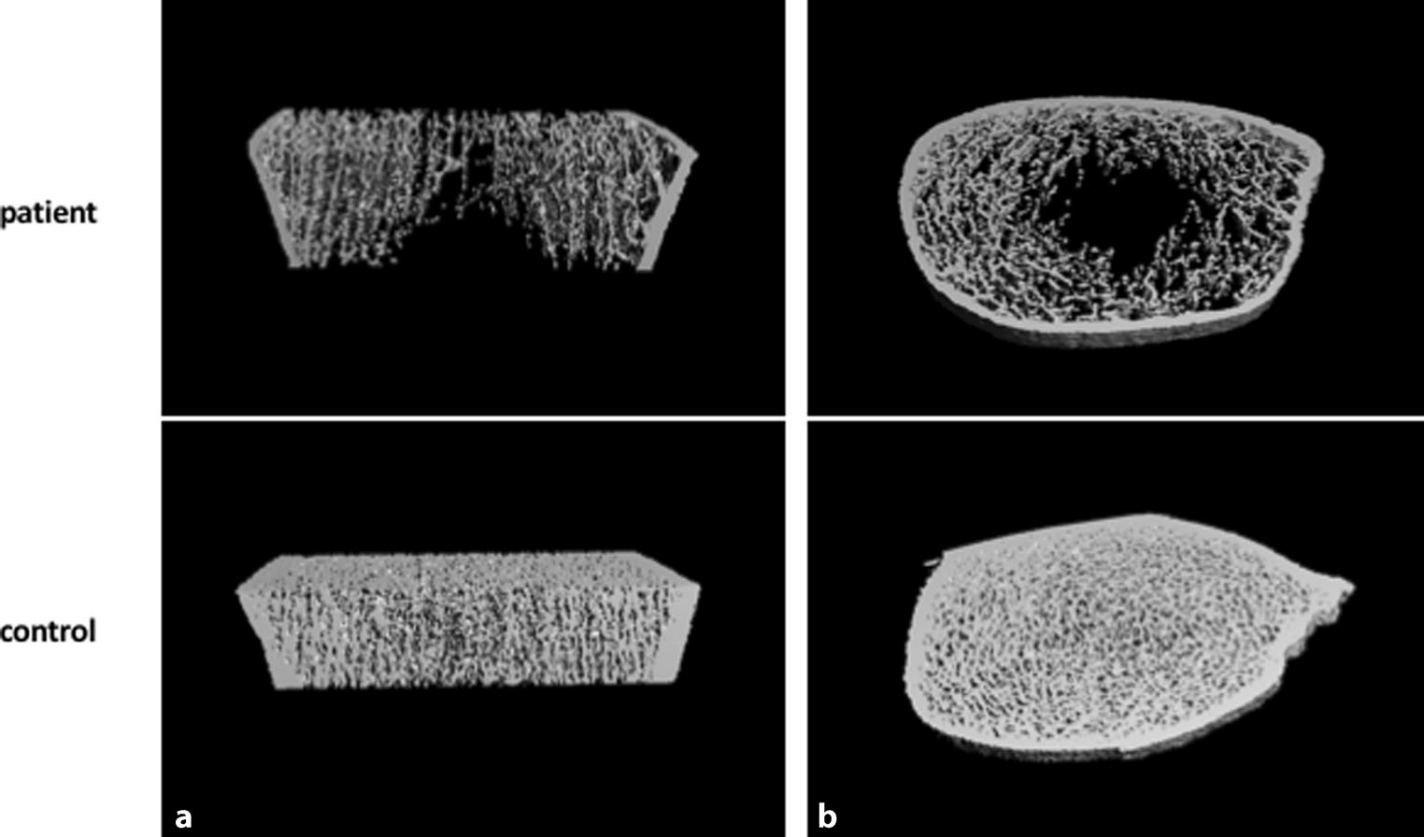
Bone microarchitectural alterations at the tibia of the patient compared to a bone with regular bone microstructure. 3D-reconstruction in coronal view (**a**) and axial view (**b**)

A dual-energy x‑ray absorptiometry (DXA) bone densitometry revealed a Z‑score of less than *−*2 reflecting a diminished age-adjusted BMD (see Table 1). High-resolution peripheral quantitative computed tomography (HR-pQCT, Scanco, Bruettisellen, Switzerland) was performed according to the manufacturer’s recommendations on calibration and scanning procedures. With an in vivo resolution of 82 μm, HR-pQCT was used to noninvasively assess volumetric bone mineral density and bone microarchitecture at the distal tibia and ultradistal radius. At the tibia (Fig. 1) normal cortical vBMD but very low total vBMD and trabecular vBMD were observed. The trabecular bone volume (BV/TV), number of trabeculae (Tb.N) and the trabecular thickness (Tb.Th) were also decreased. In addition, a high level of inhomogeneity of trabeculae was shown by this measurement. Additionally, the cortical thickness was decreased (Table 1).

**Fig. 2:**
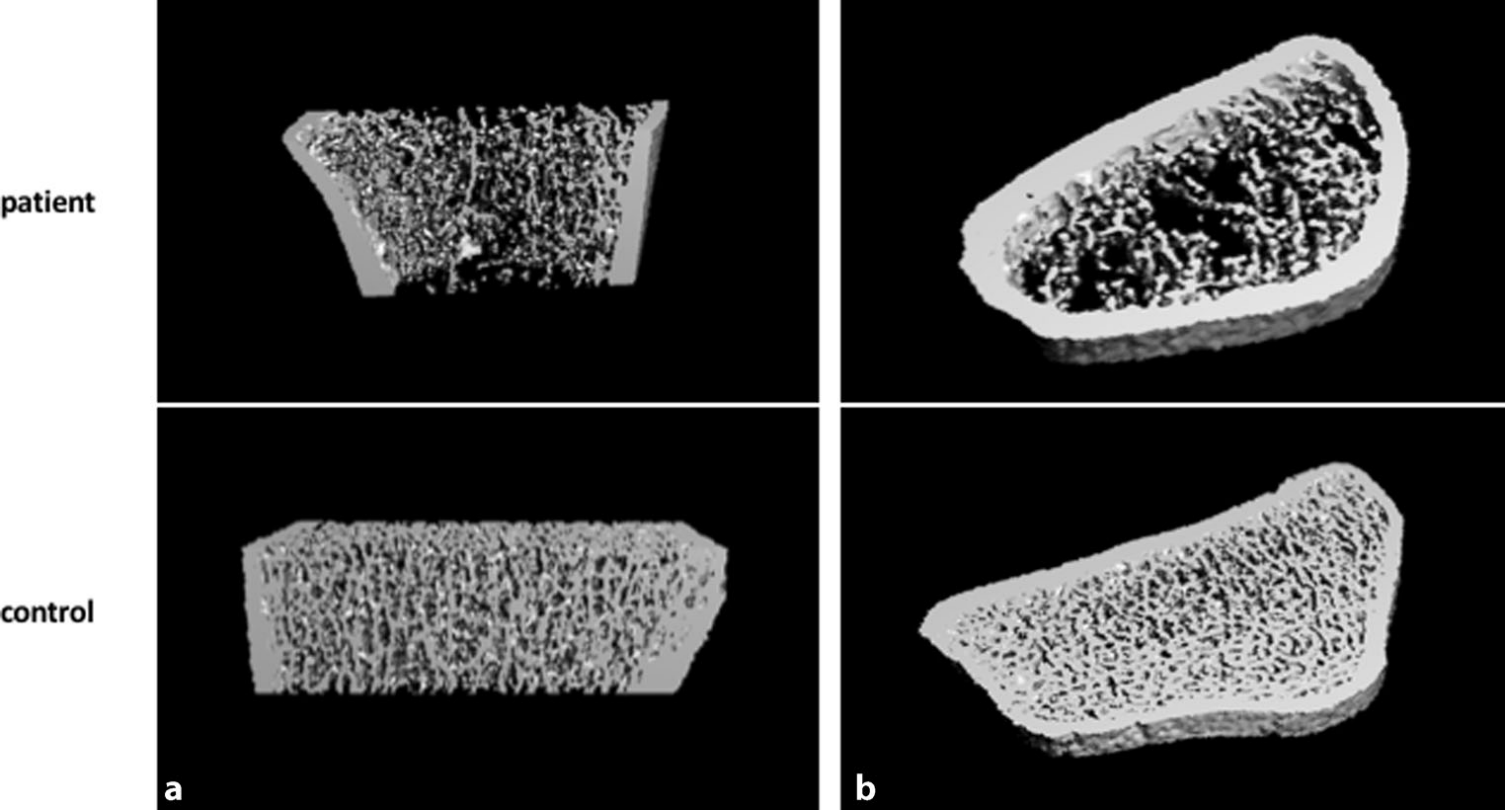
Bone microarchitectural alterations at the radius of the patient compared to a bone with regular bone microstructure. 3D-reconstruction in coronal view (**a**) and axial view (**b**)

At the radius (Fig. 2) as a non-weight-bearing bone site, similar alterations in trabecular bone were observed. In contrast the cortical vBMD was slightly increased and cortical thickness as well as total vBMD were within normal range (Table 1).

Due to 25-hydroxyvitamin D insufficiency and the deterioration of bone microarchitecture in conjunction with a fragility fracture, a supplementation with cholecalciferol and calcium as well as a primary osteoanabolic treatment with teriparatide was initiated. After 9 months of osteoanabolic therapy a combination with denosumab as an antiresorptive treatment is planned. Close follow-up and aftercare programs were arranged on the patient’s behalf. The patient was informed that data concerning the case would be submitted for publication and she provided written consent.

## Discussion

We report on a young woman with a genetically proven heterozygous form of beta and alpha thalassemia who had sustained a low-trauma fracture of the inferior pubic ramus. Among normal markers of bone metabolism, significantly decreased values of trabecular volumetric BMD and clearly reduced bone microarchitecture at the time point of peak bone mass were found.

A number of genetic and acquired factors that affect bone density in patients with thalassemia have already been detailed [6]. Toumba et al. describe the mechanism of pathogenesis in thalassemia major as multifactorial. Lack of sex steroids and impaired growth hormone in thalassemic patients due to pituitary damage, as well as other endocrine complications such as hypoparathyroidism and vitamin D deficiency contribute to failure of achieving optimal peak bone mass [4, 5]. In more severe forms, thalassemia leads to ineffective erythropoiesis and requires transfusion therapy. Chronic treatment with transfusions can result in severe iron overload in multiple organs. Subsequently, iron toxicity and other factors such as bone marrow expansion, hypogonadism and increased bone turnover are some of the many causes that are associated with BMD decrease. Patients with transfusion-dependent thalassemia have a significant decrease in BMD at the femoral neck and the total body. However, high hemoglobin, as aspired to, is attended by an increase in lumbar spine in these patients [3].

Persons with thalassemia minor (heterozygote form) are usually symptomless, but the peripheral blood picture reveals minimal anemia and microcytosis. However, thalassemia minor was also described to be a risk factor for osteoporosis resulting in low BMD [8].

Another study reported that in vivo neutron activation analysis to measure hand-bone phosphorus, single-photon absorptiometry to measure forearm bone mineral content and DXA to measure spinal BMD did not show any significant differences between a thalassemia minor group and a healthy control group. An investigation concerning markers of bone metabolism did not show any differences between thalassemia and healthy subjects, as has been found in our patient [7]. Currently, there are no reported investigations regarding bone architecture/microstructure in thalassemia.

Whereas our patient does not suffer from any eating disorder such as bulimia and anorexia, she had a low BMI. Previous studies showed that low BMI can be a risk factor especially for low trauma hip fractures, but is not associated with lower leg fractures and distal forearm fractures [9]. Furthermore the severe alterations as shown in HR-pQCT cannot be solely explained by low body weight.

Because of the young age of the patient at the time point close to the peak bone mass and the severe deteriorations of trabecular bone microstructure in conjunction with a fragility fracture, an osteoanabolic treatment with teriparatide (TPTD) was initiated to stimulate osteoblast activation and to immediately increase bone formation. We will administer—based on the medical history and the non-invasive findings— a combination therapy with an antiresorptive drug after 9 months of ongoing TPTD monotherapy to achieve a reopening of the anabolic window in order to optimize the anabolic effects of TPTD treatment [10, 11].

Due to the patient’s young age and the advantageous results concerning BMD and bone microarchitecture in previously mentioned combination therapy studies, we propose to initiate denosumab (60 mg every 6 months subcutaneously) as an anticatabolic therapeutic agent after 9 months of teriparatide monotherapy to maximize bone formation and secondary mineralization [12].

In conclusion, we report on a young patient with a heterozygous beta and alpha thalassemia and a low BMI, who had sustained a fragility fracture. Despite normal markers of bone metabolism, highly decreased values of trabecular volumetric BMD and massively reduced bone microarchitecture were found. Based on our findings, alterations in bone microarchitecture and consecutively increased fragility fracture risk should be taken into account in heterozygous beta and alpha thalassemia patients with or without a low BMI.
